# Extraction of Rhenium and Osmium from Lead Technogenic Raw Materials of Copper Production

**DOI:** 10.3390/ma15124071

**Published:** 2022-06-08

**Authors:** Berdikulova Feruza, Zharmenov Abdurassul, Terlikbaeva Alma, Sydykov Alimgazy, Serikbayeva Akmaral

**Affiliations:** RSE “National Center on Complex Processing of Mineral Raw Materials of the Republic of Kazakhstan”, Almaty 050036, Kazakhstan; jarmen56@mail.ru (Z.A.); alma_terlikbaeva@mail.ru (T.A.); depart-science@mail.ru (S.A.); akm_rgp@mail.ru (S.A.)

**Keywords:** rhenium, osmium, lead sludge, metal recovery, black lead, rhenium-containing slag

## Abstract

Lead sludge from copper production is a source of rare metals, such as rhenium and osmium, whose content reaches 0.06–0.08% and 0.0025–0.0050%, respectively. The base of the sludge consists of lead sulfate. A method of reductive smelting of lead sludge from copper smelting production at 1000–1100 °C has been developed. Coke was used as a reducing agent and sodium sulfate as a slag-forming material. Optimal conditions for selective extraction of rare metals in smelting products were found: osmium in the form of metallic form into raw lead and rhenium in the form of perrhenate compound Na_5_ReO_6_ into sodium-sulfate slag. The developed technology makes it possible to extract rhenium with a high degree of extraction in the form of water-soluble compounds for the subsequent production of commercial salts of rhenium by the known hydrometallurgical methods. The content of rhenium in the slag phase is 0.18–0.25%, with its initial content in the slime of 0.06–0.08%. The degree of rhenium concentration at the first stage of processing is 3–3.2 times in the form of water-soluble perrhenate. Osmium and lead do not form solid solutions; osmium in crude lead is mainly concentrated in the lower zones of lead. A method of obtaining a concentrate containing 53–67% osmium from raw lead with an initial content of 0.0025–0.0050% in the slurry and a concentration number of 13,000–21,000 times has been proposed.

## 1. Introduction

The object of this study is lead sludge of the wet dust collection system of copper production. Rhenium and osmium as siderophile-chalcophile elements are found in sulfide copper ores. Copper ores and concentrates of Central Kazakhstan contain rhenium and osmium-187, formed as a result of the radioactive decay of rhenium-187 [1]:

Re^187^_75_ → Os^187^_76_ + β (1)

During reduction smelting of copper concentrating in reflection or electric furnaces, rhenium is volatilized by 75–80% and osmium almost completely remains in the matte. During further matte conversion, rhenium and osmium are oxidized and concentrated in converter gases and dust. Converter gases are combined with electric furnace gases and sent to the wet gas cleaning system, where lead sludge is formed [2]. Lead slime contains up to 800 g/t of rhenium and 50 g/t of osmium and is regarded as a promising raw material for rare metals. The basis of lead sludge is lead sulphate, with lead oxide and lead sulphide present in small amounts up to 1.5%. The developed methods and technologies according to the principles of their processing can be divided into the following categories:

- Oxidative roasting or oxidative leaching with transfer of osmium in the gas phase of rhenium in solution [3,4,5,6,7,8];
- Dissolution of the main component of the sludge—lead sulfate and concentration of rare metals in the insoluble residue [9,10,11,12].

All of the above-mentioned scientific developments are aimed at increasing the degree of extraction of rare metals and obtaining concentrates with the maximum concentration number and content of rare metals. Further extraction of rare metals of commercial quality is proposed by traditional methods of processing: osmium by reduction of dioxide to metallic state, obtaining ammonium perrhenate by extraction–extraction methods or sorption-desorption from leaching solutions.

The above developments have the following disadvantages:

- Oxidation of osmium and rhenium occurs during oxidation roasting of raw materials and their collective transition into substrates, which requires their separation at the next stages of processing, and also in substrates’ concentrate toxic elements such as arsenic and mercury contaminating osmium and rhenium containing intermediate products;
- The conversion of lead sulfate into solution requires huge amounts of saturated sodium chloride solutions, the subsequent regeneration of which requires significant capital expenditures. Moreover, a product of processing is the collective concentrate of osmium and rhenium.

Based on the physicochemical properties of osmium–chalcophiles, there is an accumulating ability in melts of lead, silver and copper in the reduction processes of oxides of these metals [13,14] and high affinity for oxygen even in weak reducing atmospheres with the formation of rhenium oxide [15]. Moreover, with the main component of lead sludge in the form of lead sulfate, we set the goal of studying the process of reduction smelting with the production of black lead. Sodium sulfate was used as a slag-forming material to preserve the anion of the same name in the system. The use of sodium sulfate in the processes of smelting lead raw materials was studied in [16].

## 2. Materials and Methods

Lead sludge of the following chemical composition was taken for the study, %: 56.5–62.5 Pb; 0.21–0.47 Cu; 0.03–0.19 Zn; 0.43–1.20 Hg; 0.10–0.30 As; 0.08–0.18 Se; 0.10–0.18 Fe; 0.03–0.04 Bi; 0.06–0.07 Cd; 0.01–0.02 mg; 0.0060–0.0073 Ag; 0.0600–0.0840 Re; 0.0020–0.0050 Os; 3–5% organic.

The chemical composition of the slurry, except for rhenium and osmium, was carried out on an atomic absorption spectrometer (AAS, Agilent 240AA, Agilent Technologies, Santa Clara, CA, USA). Osmium and rhenium contents were determined by photocolorimetry methods [15,17]. The chemical composition of the reduction smelting products was additionally analyzed by X-ray spectroscopy (XSM, JCXA 733, «Superprobe», JEOL, Japan) and electron-probe microanalysis on a micro probe using an energy dispersive spectrometer (EDS, INCA ENERGY, Company “Oxford Instruments Analytical”, Great Britain) at an accelerating voltage of 15 kV, probe current 25 nA.

Anhydrous sodium sulfate containing 98% of the main substance used as a slag-forming material corresponds to the “chemically pure” grade.

As a reducing agent, we used a special coke from the Shubarkul coal deposit, which has the following technical characteristics: ash content—10–15%; volatile matter yield—2–3%; mass fraction of total sulfur—0.4 %; mass fraction of total phosphorus—0.02%; solidity—60–65%; reactivity—5–8 mL/g·s; porosity—33–37%.

**Figure 1:**
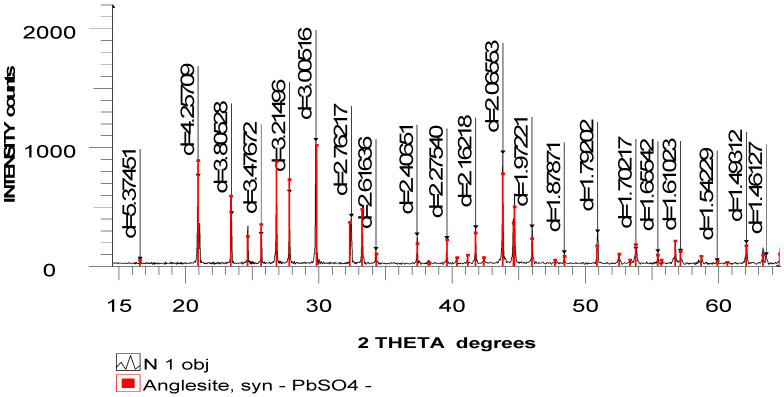
X-ray of lead sludge.

X-ray phase analysis demonstrated that the lead slurry is almost 100% lead sulfate, as shown in Figure 1. The analysis was carried out on an automatic diffractometer DRON-3 with CuKa-radiation and a β-filter. Conditions of diffractograms: U = 35 kV; I = 20 mA; firing = θ–2θ; detector = 2 deg/min.

X-ray phase analysis on a semi-quantitative basis was carried out on the basis of diffractograms of powder samples by the method of equal weights and artificial mixtures. Quantitative ratios of crystalline phases were determined. Diffractograms were interpreted using data from the ICDD: Powder Diffractometer Database PDF2 (Powder Diffraction File) Release 2022 and mineral diffractograms without impurities.

Quantitative analysis for lead forms was performed by chemical analysis methods (Table 1).

Thermogravimetric studies were carried out on the MOM derivatograph (OD-102, Budapest, Hungary). The used method is based on registration of changes in thermochemical and physical parameters of the substance, which can be caused by its heating. The thermochemical state of the sample is described by curves: temperature (T), differential thermoanalytical (DTA), thermogravimetric (TG) and differential thermogravimetric (DTG), the latter curve being a derivative of the TG-function. The analysis was performed in air, in the temperature range from 20 to 1000 °C.

Furnace heating mode is linear (dT/dt = 10), the reference was calcined Al_2_O_3_. For the uniqueness of firing conditions’ weight of samples, No. 1, 2, 3 and 4 was strictly 200 mg, with sensitivity of scales at 50 mg. The analysis was carried out within the following instrumentation limits: DTA = 250 μV, DTG = 500 μV, TG = 500 μV and T = 500 μV.

The procedure of reduction smelting: Experiments were conducted with 100 g of lead sludge; the consumption of sodium sulfate and coke was calculated by the mass of the sludge. Carefully mixed components of the charge of the studied composition: sludge, sodium sulfate and coke are placed in an alundumina crucible, which is then moved into a shaft furnace with saltpetre heaters. The furnace temperature is raised to the 1000–1100 °C and the temperature is incubated at the target temperature. The reaction zone temperature is measured with a platinum–platinum–rhodium thermocouple. The furnace is equipped with a gas recuperation system. After completion of the reduction smelting process, the furnace is turned off and cooled to room temperature, and the crucible is broken and smelting products of blackening and slag are separated by phase boundaries, then weighed and analyzed for metal content. Residue and the gas phase are precipitated in sodium sulfide-alkali solutions of 5% sodium sulfide solution in 20% caustic soda solution. The choice of the composition of the absorption solutions is aimed at the completeness of absorption of the sublimed metals in the form of sulfide and/or hydroxide precipitates.

## 3. Results

### 3.1. Results of Reduction Smelting

The process of reduction smelting in the temperature range of 1000–1200 °C, duration of 20–60 min and the consumption of sodium sulfate 10–40% and coke 2.5–7.5% was studied. The study of the effect of sodium sulfate and coke consumption was carried out at a temperature of 1100 °C with a duration of 60 min, as, according to preliminary experiments under these conditions, a clear separation of boundaries between soot and slag was observed.

**Figure 2:**
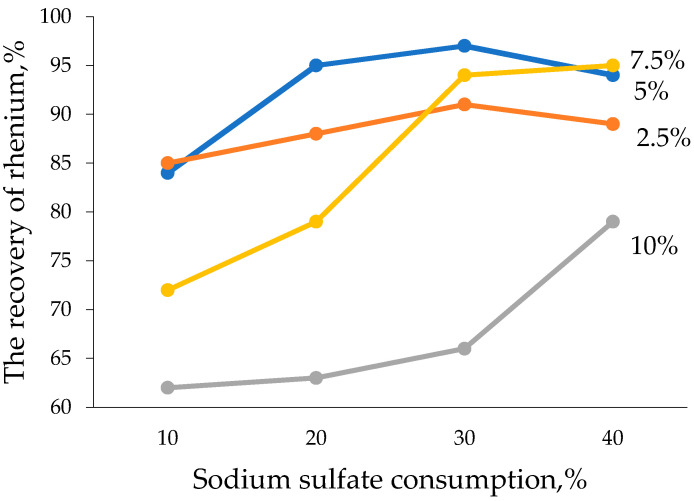
Effect of consumption of sodium sulfate and coke on the degree of extraction of rhenium into slag.

**Figure 3:**
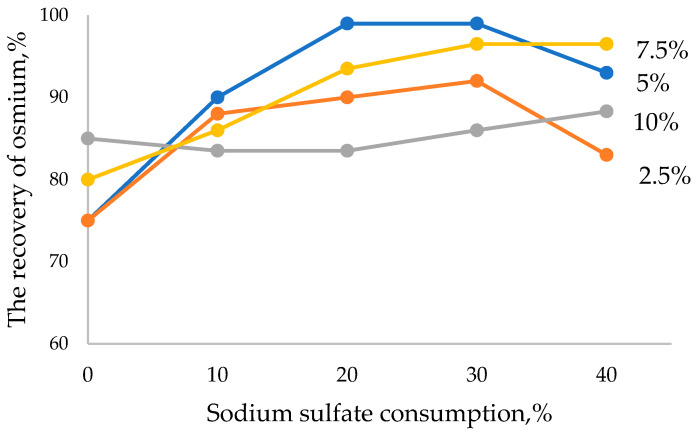
Effect of consumption of sodium sulfate and coke on the degree of extraction of osmium into the alloy.

The results of the experiments demonstrated that during reduction, with the smelting of lead slime with sodium sulfate 30% and coke 5% consumption (100 lead slime: 30; sodium sulfates: 5 coke), 97% of rhenium goes to the slag phase and 99.5% of osmium goes to raw lead, i.e., selective separation of rhenium and osmium is realized on the first stage of raw material processing, as shown in Figure 2 and Figure 3. The weight of raw lead is 52.5 g with 0.005% osmium content and 28.2 g slag with 0.23% rhenium content.

Reduction of coke consumption down to 2.5% with the consumption of 30% sodium sulfate leads to the reduction of rhenium recovery into slag (90%) and osmium into crude lead (91%), and the recovery of lead into crude lead does not exceed 80%. The presence of osmium and rhenium in subsoil under these conditions indicates the insufficiency of the reducing agent in the charge, which led to volatilization of rare metals in the form of higher oxides, OsO_4_ and Re_2_O_7_. At a reduction of sodium sulfate consumption to 10% with coke consumption of 5%, a reduction of rare metal extraction into the smelting products is also observed. This is due to the formation of sodium sulfide in the system by increasing the ratio of Na_2_SO_4_:C and increasing the viscosity of the slag phase, retaining part of the metal fractions. Moreover, these data show that the sodium sulfide melt is not able to trap rhenium.

The increase in sulfide sulfur content in the melt demonstrates the chalcophile properties of osmium with its subsequent concentration in the matte phase, which is formed at the ratio of 100 lead slurry of 10Na_2_SO_4_:5C. At the optimum composition of the charge of 100 lead slurry 30Na_2_SO_4_:5C, only crude lead and slag are formed with a clear separation of the interface. Increasing the consumption of sodium sulfate also reduces the degree of extraction of osmium in the raw lead; in these conditions, part of it goes to the gas phase.

Increasing the temperature of reduction smelting to 1200 °C reduces the duration of the process to 10–20 min, because with increasing temperature the diffusion rate of metals increases and the equilibrium of the system is instantly established. In the reduction smelting of lead slime with sodium sulfate consumption of 30% and coke consumption of 5% at 1100 °C and a duration of 30–60 min, the highest separation rates of osmium and rhenium into smelting products and the degree of their extraction were achieved. The formed slag mainly consists of sodium sulfate and is water-soluble; rhenium completely passes into the solution, which indicates its concentration in the slag in the form of perrhenate compounds.

In Table 2, the distribution of nonferrous metals in the final smelting products is studied. Lead goes to rough lead (95–96%), and more than 99% of osmium and bismuth. In the slag, mainly rhenium and arsenic is released. Mercury, partly arsenic, cadmium and selenium are transferred to sublimate.

### 3.2. Thermogravimetric Studies

**Figure 4:**
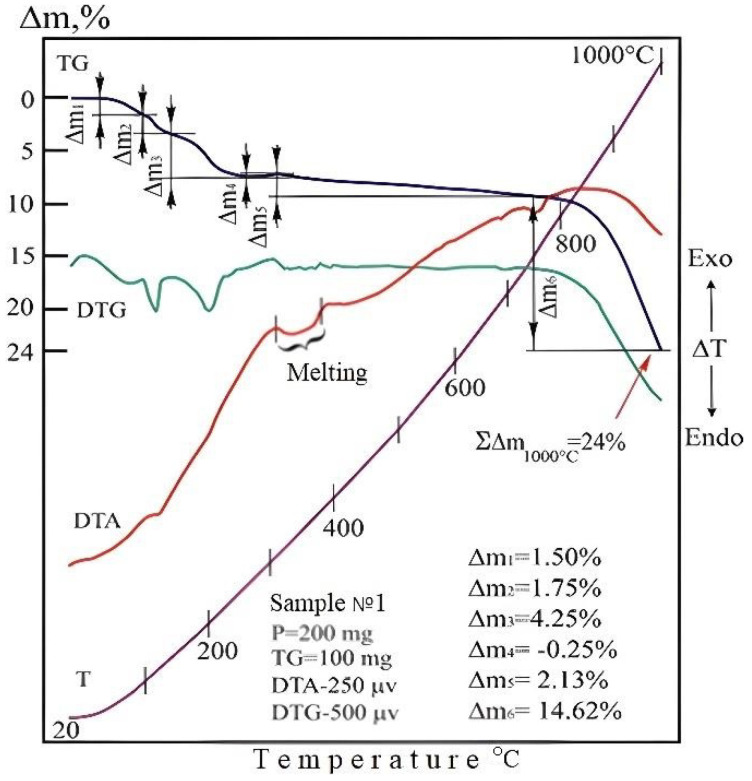
Thermogravimetric readings of lead sludge at 20–1000 °C with 40–85 °C change in sample mass: Δm_1_ = −1.50%, -H_2_O; at 85–155 °C; -Δm_2_= −1.75%, -H_2_O; at 155–240 °C—Δm_3_ = −4.25%, -CO_2_; -H_2_O; at 240–320 °C—Δm4 = +0.25% +O_2_; at 320–430 °C,—Δm_5_= −2.13%, -CO_2_; at 740–1000 °C—Δm_6_ = −14.62%—SO_2_, sublimation; at 20–1000 °C sample mass change in total Δm = −24.00%.

For thermogravimetric study of the reductive smelting process of the charge, first of all, thermogravimetric analysis of the lead slurry was carried out. In the temperature range of 40–240 °C, there is a process of dehydration and removal of CO_2_ from the system as a result of the decomposition of organic substances. The 240–430 °C lead slurry firing, the oxidation reactions of osmium and rhenium, does not occur, despite the introduction of oxygen into the reaction zone. At 350–370 °C, the melting of some of the organic compounds present in the lead sludge is observed, followed by decomposition. Further, when the temperature is raised to 700 °C, the transition of osmium and rhenium into the gas phase is only 68% and 32.5%, respectively. When the temperature rises above 700 °C, partial melting of lead slime begins. This explains the inhibition of osmium and rhenium release into the gas phase as a result of formation of a thin crust in the charge. At a further increase in the temperature of calcination (800–1000 °C), the trajectories DTA-, DTG- and TG-lines of the specified objects with identical speed rush downwards horizontally, describing the process of sublimation of a residual product of calcination, as shown in Figure 4. 

**Figure 5:**
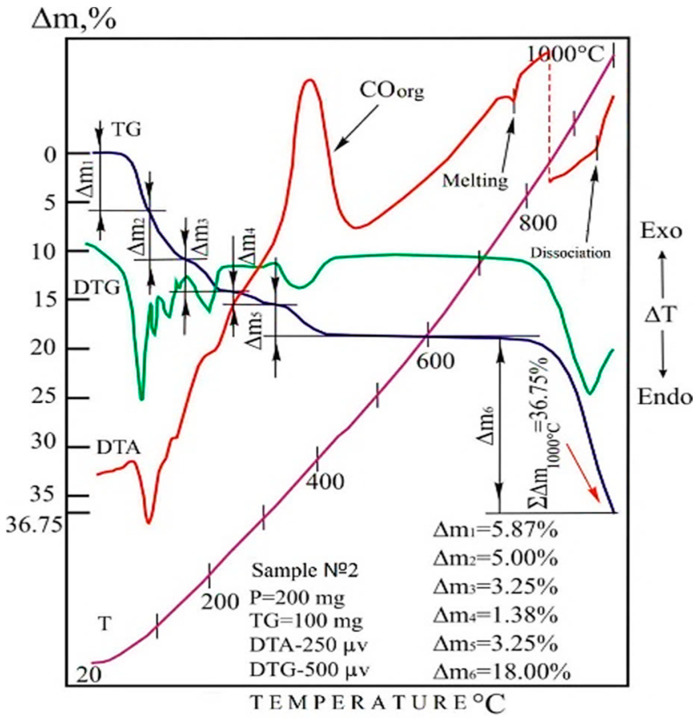
Thermogravimetric readings of the charge 100 lead sludge: 30 sodium sulfates; 5 cokes at 20–1000 °C at 40–85 °C change in sample mass Δm_1_ = −5.87% -H_2_O; at 85–155 °C—Δm_2_ = −5% -H_2_O; at 155–240 °C—Δm_3_= −3.25% -CO_2_; -H_2_O; at 240–320 °C—Δm_4_ = 1.38 -CO_2_; -SO_2_; at 320–430 °C—Δm_5_ = −3.25% -CO_2_; at 740–1000 °C—Δm_6_= −18%—SO_2_, sublimation; and at 20–1000 °C sample mass change in total Δm= −36.75%.

Thermogravimetric analysis of the sample, including the lead slurry, sodium sulfate and coke at a linear increase in temperature from 20 to 1000 °C, left a number of effects on its derefinial and thermogravimetric curves associated with the decomposition reactions of sample components, as shown in Figure 5. The initial phase of thermal dissociation of the powder mixture proceeds up to 240 °C. Between 20–85 and 85–155 °C, this process is represented by a two-stage dehydration of the system, in which the test sample decreases in mass by 5.87 and 5.0%, respectively (Δm_1_ and Δm_2_). Then (in the interval 155–240 °C) the loss of the rest of the water is accompanied by emissions of sulfur dioxide, the sources of which are elementary sulfur in the lead sludge. The total amount of gases (Δm_3_), withdrawn from the system in the form of H_2_O and SO_2_ at these temperature intervals, was 3.25% of the mass of the test sample [17,18].

Judging by the morphology of the DTA and DTG curves in the 240–320 °C interval, the linear slope of the thermogravimetric line in this temperature range is due to the joint removal of the sulfurous part of the sodium complex (SO_2_) and part of the CO_2_ generated by coke at the initial stage of its oxidation from the sample. The mass loss within these temperatures corresponds to 1.38% of the mass of the analyzed sample.

The next stage of the thermal transformation of the test mixture takes place in the 320–430 °C range. The powerful exothermic effect described by the DTA curve at 380 °C is the result of the oxidation cycle of coke CO_org_ to CO_2_. The reaction is accompanied by intense release of synthesis gas into the atmosphere, leaving a clearly expressed step of mass loss on the TG curve and a fully developed peak on the DTG line, reflecting the kinetics of volatile carbon dioxide formation.

The thermal degradation of the sample is entirely completed by the dissociation of lead sulfate. This reaction starts in the region of 740 °C and ends at 1000 °C. This process is due to the melting of the mixture of lead sulfate and sodium sulfate, which left a pronounced endothermic peak on the DTA curve (740–790 °C).

According to [19], the thermal decomposition of lead sulfate begins at 840 °C; in our case, the early decomposition of lead sulfate can be explained by the influence of coke and sodium sulfate, which weaken the Pb-SO_4_ bonds. Those decomposition and reduction reactions begin at 740 °C, as follows [20]:

PbSO_4_ → PbO·PbSO_4_ → 2PbO·PbSO_4_ → 3PbO·PbSO_4_ → PbO (2)

at 720 °C PbS + 7PbSO_4_ → 4(PbO·PbSO_4_) + 4SO_2_ (3)

at 863 °C PbS + 10(PbO·PbSO_4_) = 7(2PbO·PbSO_4_) + 4SO_2_ (4)

at 910 °C PbS + 2PbO·PbSO_4_ = 5Pb + 3SO_2_ (5)

at 930 °C 3(2PbO·PbSO_4_) + SO_2_ = 4(PbO·PbSO_4_) + Pb (6)

at 965 °C PbO·PbSO_4_ + Pb = PbO + SO_2_ (7)

at 1080 °C PbSO_4_ → PbO + SO_3_ (8)

In parallel with these reactions, the following reactions occur, as the reduction of lead sulfate with carbon monoxide formed in the system [21]:

PbSO_4_ + 4CO(g) = PbS + 4CO_2_(g) (9)

PbSO_4_ = 1/2PbO·PbSO4 + 1/2SO_2_(g) + 1/4O_2_(g) (10)

PbSO_4_ + 1/2CO(g) = 1/2PbO·PbSO_4_ + 1/2SO_2_(g) + 1/2CO_2_(g) (11)

PbSO_4_ + CO(g) = SO_2_(g)+ CO_2_(g)+ PbO (12)

PbO + CO(g) = Pb + CO_2_(g) (13)

PbSO_4_ + 1/3PbS = 4/3PbO + 4/3SO_2_(g) (14)

In the molten state, the system under study (Pb-SO_4_) forms weak bonds, which break when the temperature increases to form more stable compounds, such as SO_2_. The latter easily leave their former positions and rush into the atmosphere, facilitating the product of firing by more than 18%.

The obtained thermogravimetric readings of the system under the test, taking into account the mass loss beyond 1000 °C, are in good agreement with the set of stoichiometric data related to the components of the analyzed mixture.

Thermogravimetric studies and the results of physical and chemical analyses of crude lead and slag, consisting mainly of sodium sulfate, demonstrate that the reduction smelting of lead slime, with a sodium sulfate consumption of 30% and a coke consumption of 5%, proceeds with the formation of crude lead due to the decomposition of lead sulfate with a subsequent reduction to metallic lead. Osmium compounds are also reduced to a metallic state and collected in the lead melt, like all platinum metals [22].

**Figure 6:**
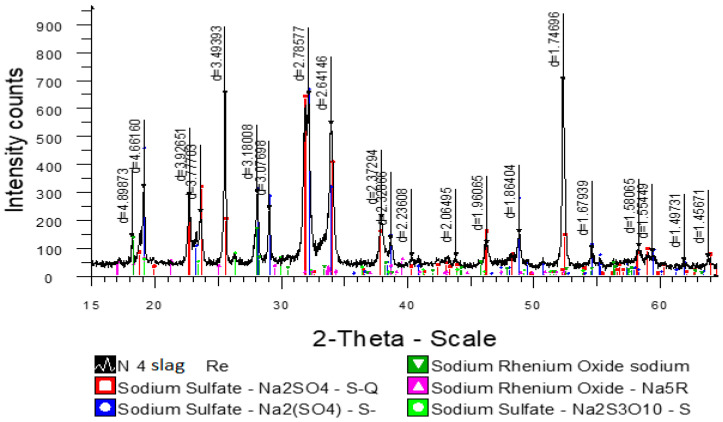
Diffraction pattern of sodium sulfate slag.

Sodium sulfate serves as a slag-forming element, where elements with high affinity to oxygen, such as rhenium and arsenic, are concentrated to form perrhenates and arsenates. The X-ray diffraction analysis of sodium sulfate slag at Figure 6 shows that sodium sulfate is present in two structural modifications, part of the sodium sulfate (41.4%) also undergoes structural changes during melting, and rhenium is present in two perrhenate forms, NaReO_4_ and Na_5_ReO_6_. Formation of perrhenates with anionic residue ReO^4−^ proceeds in wide temperature intervals with metal oxides, but Na_5_ReO_6_ formation requires special conditions of synthesis at the interaction of sodium oxide Na_2_O and metallic rhenium [22].

These data also do not exclude the formation of metallic rhenium with the subsequent formation of the compounds, Na_5_ReO_6_. Arsenic in the slag is concentrated in the form of lead arsenic at Table 3. When dissolving slag in water S:L =1:3–5, at 70 °C rhenium completely passes into solution, arsenic remains in an insoluble residue, and the yield of insoluble residue is 0.5–0.7%. After cooling, the solution is recrystallized with sodium sulfate and the productive rhenium solution is filtered. A solution of the following chemical composition, g/L, was obtained: 5.3-Na, 2.4-Pb, 0.1-As, 0.2-Cu, 0.08-Se, 0.09-Zn, 0.2-I, and 4.24-Re.

From the given solution, rhenium can be extracted by sorption–desorption methods on ions [23] or on a sorbent obtained from rice husk [24], precipitation with ammonium salts [25], and also by extraction–re-extraction. In our case, rhenium was extracted in the form of ammonium perrhenate corresponding to AP-00 grade by extraction and re-extraction. Recrystallized sodium sulfate contains up to 0.005% rhenium, which is returned to the lead slurry smelting process.

The degree of extraction of rhenium into ammonium perrhenate is not less than 85%, because the first stage provides a high degree of extraction and separation of rhenium from interfering elements for the next stages of commercial products, compared with known methods (Table 4).

Sodium Sulfate Na_2_SO_4_—52.4%; Sodium Sulfate Na_2_(SO_4_)—41.4%; Sodium Rhenium Oxide sodium perrhenate NaReO_4_—0.28%, Sodium Rhenium Oxide Na_5_ReO_6_—0.20%; Sodium Sulfate Na_2_S_3_O_10_—1.5%.

Physicochemical studies of black lead collecting osmium demonstrated uneven distribution of osmium in sections; the main amount is concentrated in the lower parts of the melt. Osmium and lead do not form solid solutions; osmium in the melt is concentrated in the form of individual metal droplets, due to which an uneven distribution of osmium is observed.

Three samples of lead niello (lead melt) obtained by reduction smelting of lead slime containing different amounts of osmium have been studied: slime No. 1 contained 0.0025% osmium, No. 2—0.0035% osmium, and No. 3—0.0055% osmium. The rough lead obtained from these slurries was conventionally divided into three parts according to the height of the upper part, the middle part, and the lower part. In the upper part of the lead, in all samples, the osmium was not detected; its main amount was determined in the lower parts, with the content of osmium in the middle part being within 0.0008–0.002%, and in the lower part 0.05–0.09% (Table 5).

In this case, the degree of osmium concentration in the lower parts of the lead is directly proportional to the osmium content in the initial lead slurry; this is possible due to the formation of larger conglomerates of osmium in the lead melt with its significant content in the raw material. The concentration of osmium in the lower parts of the melt is explained by the difference in density of metals; the density of osmium 22.2 g/cm^3^ is twice as high as the density of lead, at 11.3 g/cm^3^. Similar results were obtained in [23].

**Figure 7:**
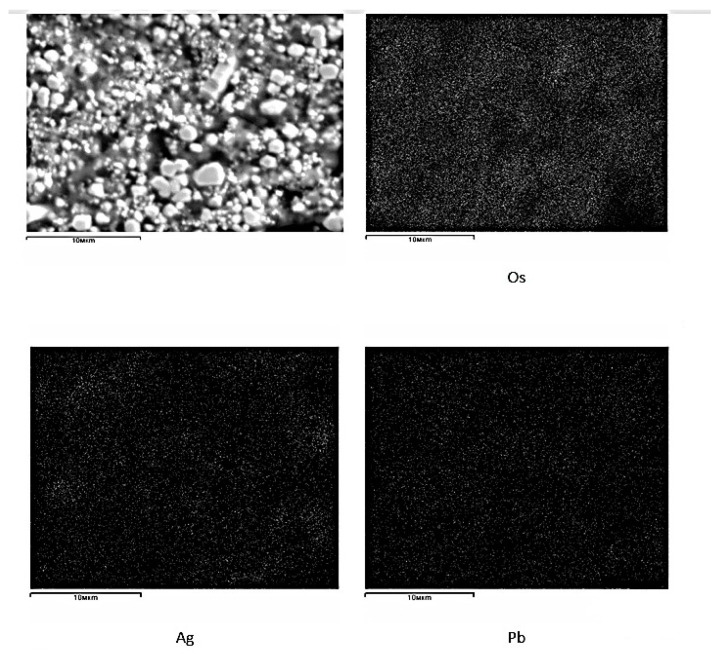
Microstructure and distribution density of elements in the concentrate.

To study the forms of finding osmium, concentrated parts of crude lead were crushed and dissolved with nitric acid with a concentration of 8% at S:L=1:3 at 60 °C to transfer lead into the solution. Under these conditions, no transition of osmium to solution or to the gas phase was observed. Two-fold dissolution yielded a concentrate containing 65% osmium and 15% lead. Further reduction of the concentrate mass with a dissolution of lead leads to partial oxidation of osmium; therefore, studies were conducted with this concentrate. Microprobe analysis of the concentrate in Table 6, Figure 7 shows that osmium is present in crude lead in the form of a metallic state, which indicates the completeness of its compound recovery.

The presence of lead oxides is possible because of the oxidation reaction of lead when dissolved with nitric acid. Silver, as a noble metal, is also concentrated in crude lead, but when dissolved with nitric acid, it mostly goes into the solution, and is not concentrated in an insoluble residue. The results of the research can serve as initial data for the development of a method of obtaining osmium concentrate from crude lead.

## 4. Conclusions

As a result of this research, the technology of lead sludge processing of copper production has been developed, which provides selective separation and concentration of osmium and rhenium into smelting products on the first stage of processing. Optimal conditions for reducing smelting: the composition of the charge of 100 lead sludge: 30 sodium sulfates; 5 cokes; temperature of 1000–1100 °C; process duration of 30–60 min. In reductive smelting, low-valent compounds of rhenium (ReO_2_, ReS_2_, Re_2_O_3_) oxidize to compounds Re (VII) concentrated in sodium-sulfate slag in the form of sodium-sulfate salts, NaReO_4_ and Na_5_ReO_6_. The content of rhenium in the slag phase is 0.18–0.25% with its initial content in the slime of 0.06–0.08%; the number of concentration is −3. Osmium is reduced to the metallic state and, due to the fact that it does not form solid solutions with lead, has a density of 22.3 g/dm^3^ twice that of lead; osmium is concentrated in the lower zones of lead melt. A method of nitric acid dissolution of osmium-containing parts of lead with obtaining a concentrate containing 53–67% osmium has been proposed. The degree of osmium concentration is 13,000–21,000 times. 

Rhenium-containing sodium sulfate slag is recycled by aqueous dissolution; with rhenium, more than 95% goes into the solution, and from the solution rhenium is extracted by conventional processes in the form of ammonium perrhenate. Sodium sulfate is recrystallized from the solution and serves as a slag-forming material for the following processes of lead sludge processing. 

This technology is notable for the efficiency of obtaining rhenium and osmium-containing concentrates with a high product yield and extraction degree of rare metals, as a the multisectional separation of all sludge components is ensured. Mercury, cadmium, and zinc are concentrated in subgons; the low content of other interfering elements in draft lead and sodium sulfate slag allows it to obtain high technological indicators for the extraction of rare metals from raw materials of complex composition. 

## Figures and Tables

**Table 1:** Forms of lead in lead sludge.

Lead Compounds	Mass Fraction, %
Pbgeneral	62.12–63.56
Pb (PbSO4)	53.92–61.48
Pb (PbS)	0.05–0.23
Pb (PbO)	1.51–8.80

**Table 2:** Distribution of elements in the products of reduction smelting.

Elements	Draft Lead		Slag		Sublimates	
	I	II	I	II	I	II
Pb	98.5	95.6	0.05	1.2	-	-
Cu	0.467	81.7	0.009	0.66	-	-
Zn	0.085	22.67	0.026	4.74	5–6	72.58
Na	0.05	0.001	34.55	96.0	-	-
Cd	0.037	30.36	0.002	0.6	2–2.5	69.04
As	0.025	25.83	0.193	72.75	0.01	1.92
Bi	0.067	99.2	0.005	-	-	-
Se	0.1	35.36	0.03	5.1	0.1	63.54
Hg	-	-	-	-	20–50	99.4
Os	0.005	99.5	<0.001	-	-	-
Re	0.0041	0.2	0.23	97.5	-	-

Note: I—mass content, %, II—degree of extraction, %.

**Table 3:** Results of microprobe elemental analysis of various phases of sodium sulfate slag, %.

Elements	Phases			
	1-Phase	2-Phase	3-Phase	4-Phase
Na	26.48	5.54	2.40	0.92
S	20.79	4.65	0.81	-
O	41.13	24.46	19.63	12.63
K	0.98	6.23	8.37	-
Re	3.15	52.52	62.59	-
Ca	0.37	-	0.10	0.46
Pb	3.33	1.99	1.38	68.90
As	-	-	-	14.24
Cl	1.74	-	-	2.61
Zn	-	0.77	1.31	-
Al	0.63	-	-	-
Si	1.02	-	-	-
Possible connections	Na_2_SO_4_, NaReO_4_, Na_5_ReO_6_	KReO_4_, Pb(ReO_4_)_2_	KReO_4_, NaReO_4_ Pb(ReO_4_)_2_, Na_5_ReO_6_	PbAsO_4_

**Table 4:** Comparative data of technological indicators of rhenium and osmium extraction methods.

No.	Processing Method	Technological Parameters	Disadvantages of the Method	Source
1	Sludge leaching in the presence of an oxidizer.	Rhenium extraction in solution is 95–98%; osmium extraction in the gas phase is above 95%.	High consumption of expensive oxidizing agents such as hydrogen peroxide, obtaining productive rhenium solutions of complex composition containing such impurities as Pb, Cu, Zn, As, etc.	[5,10]
2	Leaching of lead compounds with concentration of rare metals in insoluble residue.	Removal of lead sulfate in solution in the form of chloride or nitrate with a concentration of osmium and rhenium in an insoluble residue. The degree of concentration of osmium and rhenium is 20–30 times.	The consumption of solutions exceeds the weight of lead sludge by 3–5 times. Significant capital expenditures are required for the disposal of the obtained solutions.	[9,12]
3	Oxidation firing.	Extraction of osmium and rhenium in the gas phase by 95–98% with subsequent concentration in subcarbon.	There is a joint transition of osmium and rhenium in the gas phase, requiring further separation.	[3,7]
4	The method proposed by the authors.	Selective separation of osmium and rhenium at the first stage of processing. Extent of osmium extraction into blister lead—99.5%; rhenium into sodium slag—97.5%.	High temperature of 1100 °C.	

**Table 5:** Chemical composition of various sections of the lead melt, %.

No. Samples	Plots of Lead Melt	Mass, g	Pb	Cu	Zn	Bi	Os
1	Upper	124.5	94.2	1.278	0.196	0.0223	-
	Average	75.5	96.7	0.421	0.0018	0.0673	0.002
	Lower	24.7	98.3	0.23	0.0008	0.0175	0.05
2	Upper	126.6	94.3	1.190	0.1950	0.0221	-
	Average	76.2	92.5	0.678	0.0009	0.0745	0.0008
	Lower	22.4	97.9	0.289	0.0008	0.0182	0.0590
3	Upper	128.0	94.9	1.400	0.1870	0.0220	-
	Average	78.11	97.2	0.028	0.00075	0.0712	0.0008
	Lower	19.64	98.5	0.22	0.0008	0.018	0.092

Note: total mass of the melt No. 1—224.7 g; No. 2—225.2 g; No. 3—225.75 g.

**Table 6:** Results of microprobe elemental analysis of various phases of osmium concentrate, %.

Elements	Phases				
	1-Phase	2-Phase	3-Phase	4-Phase	5-Phase
Pb	51.47	4.24	41.89	1.04	30.04
Os	8.95	74.96	22.51	82.15	37.4
Ag	4.75	1.30	2.2	4.72	5.92
Se	2.32	1.94	0.71	3.02	2.94
As	-	2.96	-	3.11	-
Fe	0.75	-	0.59	-	1.03
Cr	-	0.49	0.53	-	0.7
Ca	0.32	-	-	-	-
Cl	2.17	-	0.5	-	1.46
S	9.32	1.38	8.24	-	5.38
Al	-	2.21	-	0.75	0.41
O	19.84	10.52	22.83	5.21	14.71
Possible connections	PbS, PbO, Ag_2_O, Os	PbO, Al_2_O_3_, Os, S	PbS, PbO, Ag_2_O, Os	Os, PbO, Ag_2_O,	PbS, PbO, Ag_2_O, Os

## Data Availability

Not applicable.
